# Testing in Mice the Hypothesis That Melanin Is Protective in Malaria Infections

**DOI:** 10.1371/journal.pone.0029493

**Published:** 2012-01-05

**Authors:** Michael Waisberg, Brandi K. Vickers, Stephanie B. Yager, Christina K. Lin, Susan K. Pierce

**Affiliations:** Laboratory of Immunogenetics, National Institute of Allergy and Infectious Diseases, National Institutes of Health, Rockville, Maryland, United States of America; Federal University of São Paulo, Brazil

## Abstract

Malaria has had the largest impact of any infectious disease on shaping the human genome, exerting enormous selective pressure on genes that improve survival in severe malaria infections. Modern humans originated in Africa and lost skin melanization as they migrated to temperate regions of the globe. Although it is well documented that loss of melanization improved cutaneous Vitamin D synthesis, melanin plays an evolutionary ancient role in insect immunity to malaria and in some instances melanin has been implicated to play an immunoregulatory role in vertebrates. Thus, we tested the hypothesis that melanization may be protective in malaria infections using mouse models. Congenic C57BL/6 mice that differed only in the gene encoding tyrosinase, a key enzyme in the synthesis of melanin, showed no difference in the clinical course of infection by *Plasmodium yoelii* 17XL, that causes severe anemia, *Plasmodium berghei* ANKA, that causes severe cerebral malaria or *Plasmodium chabaudi AS* that causes uncomplicated chronic disease. Moreover, neither genetic deficiencies in vitamin D synthesis nor vitamin D supplementation had an effect on survival in cerebral malaria. Taken together, these results indicate that neither melanin nor vitamin D production improve survival in severe malaria.

## Introduction

Melanins are pigments that have evolved over 500 million years and that are present in animals, microorganisms and plants. In flies and mosquitoes melanization of pathogens is part of the innate immune response and it is correlated with resistance to infection [1], [2]. In animals, melanin is produced by melanocytes, that are present in a variety of tissues, including skin, uveal tract, brain and the peritoneum [3]. The low level of melanization in the skin of humans living in temperate climates of the globe is in large part due to the fact that melanin pigments absorb and scatter the UVB wavelengths that catalyze the conversion of 7-dehydrocholesterol to pre-Vitamin D3, the precursor of Vitamin D3 in the skin [4], [5]. In fact, individuals with marked skin melanization frequently require 10–20 times longer exposure to sunlight than those of lighter pigmentation to promote adequate synthesis of vitamin D3 [4], [6]. Vitamin D deficiency can exert strong selective pressure by causing severe deformation of the pelvis in women, leading to increased perinatal morbidity and mortality, or increasing the susceptibility to infections and death in children [7]. The pressure for skin melanization in tropical regions, on the other hand, can be explained by the advantage of melanized individuals to withstand prolonged sun exposure, although a rigorous link of melanization with an increase in fitness in mammals has not been established [3]. Interestingly, dark colors are attractive to *Aedes aegyptii*, the mosquito vector of dengue and yellow fever, and *Culex fatigans*, the mosquito vector of filarial worms, but not for anopheline mosquitoes, the mosquito vectors of malaria, which find light colors more attractive [8]. In this regard skin melanization can be either advantageous or detrimental, depending on the disease.

In addition, both melanin and vitamin D have been suggested to have potential immunoregulatory functions. Melanin has been shown in some circumstances to have antioxidant [9], [10], anti-inflammatory [11], and immunoregulatory functions in innate immune responses, with melanocytes participating in phagocytosis [12] and inflammation [13], [14], [15]. This observation led to the suggestion that an additional function of melanin in the skin is to promote immune responses to infections [3], [16], [17]. In birds, melanin levels have been shown to influence the magnitude of antibody and cellular responses [18]. In addition, vitamin D deficiencies have been reported to increase [19], [20] and to decrease resistance to infection [21], [22]. Although in experiments vitamin D inhibits plasmodium growth *in vitro* [23], in malaria infections neither VDR gene polymorphisms or vitamin D levels correlate with infection [24]. On the other hand, vitamin D deficiency has been shown to be a risk factor for the development of autoimmune diseases including multiple sclerosis [25], [26], [27], [28] and systemic lupus erythematosus (SLE) [29], [30], [31]. SLE is eight to ten times more prevalent in women of African as compared to European descent and recently, we provided evidence in a mouse model of SLE that SLE susceptibility genes that result in hyperimmune activation are protective in cerebral malaria but not against severe anemia [32]. These results suggested that the higher incidence of SLE in African Americans may be a consequence of the beneficial effects of SLE susceptibility genes in malaria-endemic areas.

The possibility that melanin and vitamin D may play immunoregulatory roles led us to the hypothesis that melanin or vitamin D production may have been selected for in Africans in part due to a protective role in malaria. To test this hypothesis, we compared the course of malaria infections in wild type and albino B6(Cg)-Tyrc-2J/J mice (B6.Tyr^−/−^ mice) that have mutations in the Tyr gene, that encodes tyrosinase, the same gene that is mutated in type I human oculocutaneous albinism. Tyrosinase is the key enzyme in mammalian melanin biosynthesis that catalyzes the conversion of tyrosine to dopa, the dehydrogenation of dopa to dopaquinone, and the conversion of 5,6-dihidroxyindile to melanochrome, which is the direct precursor of melanin [33]. Consequently, the absence of tyrosinase ablates completely the synthesis of the major body melanins, pheomelanin and eumelanin. We also tested whether vitamin D had an effect on the course of malaria infections in mice.

## Results

### Melanization does not protect against malaria-induced severe anemia

**Figure 1:**
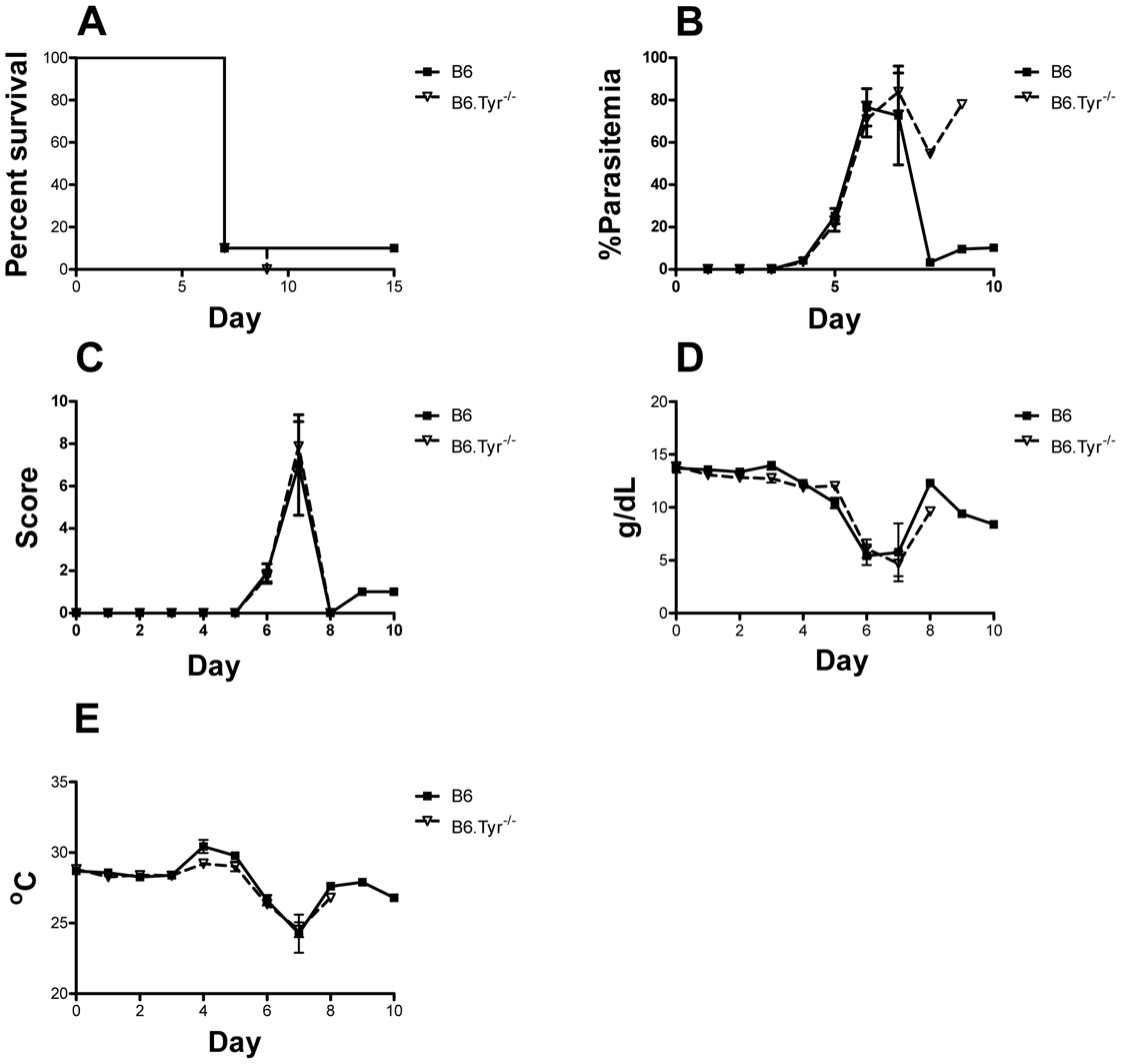
Effect of melanization in severe malaria anemia. (**A**) The percent of B6.*Tyr−/−* and B6 *P. yoelii* 17XL infected mice that survived over time is given in Kaplan-Meier curves. Death was defined as a parasitemia ≥60%, moribundity or death (P = 0.64). (**B**) Parasitemias, determined by GIEMSA stained smears of tail blood, are given for B6.*Tyr−/−* and B6 infected mice over time. (**C**) Clinical scores determined by the modified SNAP scoring system are given. (**D**) Hemoglobin concentrations of B6.*Tyr−/−* and B6 infected mice are given. (**E**) Body temperature determined at the tail skin for both B6.*Tyr−/−* and B6 infected mice over time are given. Data showed represents the mean of 10 animals and bars represent the SEM.

C57BL/6 (B6) mice and tyrosinase deficient B6 (B6.Tyr^−/−^) mice were infected with the lethal strain *P. yoelii* 17XL by injection of 10^4^ infected red blood cells i.p. Mice were followed daily to evaluate parasitemia, body weight, temperature, clinical status, hemoglobin concentration and survival (Figure 1). We also determined the hematocrit, white blood cell differential count and platelets count, on day 0 and day 3 following infection (Figure S1). Infected B6.Tyr^−/−^ mice had higher white blood cell counts, less anemia and higher lymphocyte counts on day 3 as compared to B6 mice. Despite these differences, we observed no difference in the survival of the B6 and B6.Tyr^−/−^ mice following *P. yoelii* 17XL infections. The majority of B6 and B6.Tyr^−/−^ mice died of the infection within 8–9 days and mice from the two groups showed a similar course of infection for all parameters analyzed. The levels of parasitemia appear different between the two groups after day 7 but these differences are not statistically significant as only one mouse in each group survived beyond day 7.

### Melanization does not protect against cerebral malaria

**Figure 2:**
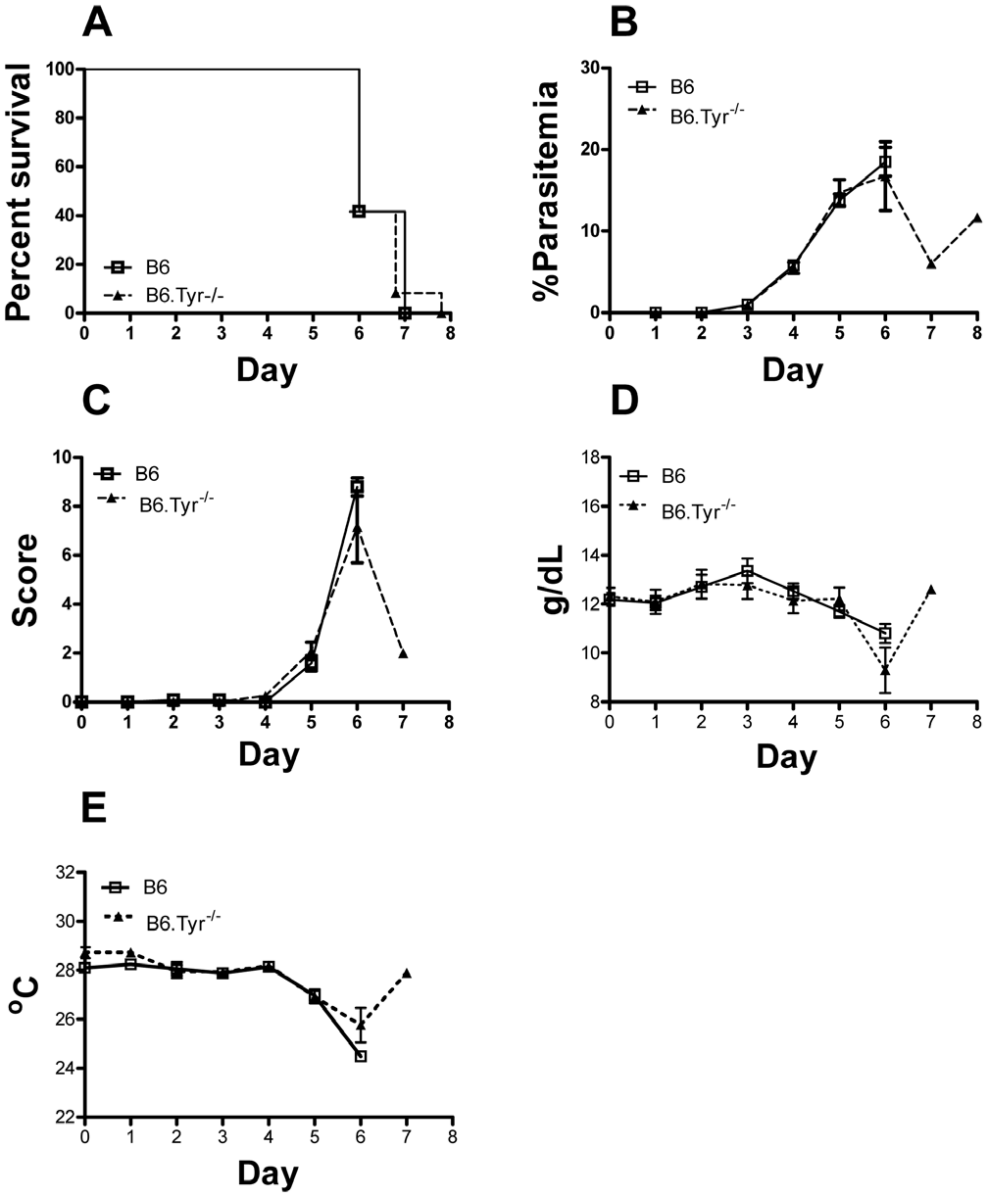
Effect of melanization on cerebral malaria. (**A**) The percent of B6.*Tyr−/−* and B6 *P. berghei* ANKA infected mice that survived over time is given in Kaplan-Meier curves. Death was defined as a parasitemia ≥60%, moribundity or death (P = 0.71). (**B**) Parasitemias, determined by GIEMSA stained smears of tail blood, are given for B6.*Tyr−/−* and B6 infected mice over time. (**C**) Clinical scores determined by the modified SNAP scoring system are given. (**D**) Hemoglobin concentrations of B6.*Tyr−/−* and B6 infected mice are given. (**E**) Body temperature determined at the tail skin for both B6.*Tyr−/−* and B6 infected mice over time are given. Data showed represents the mean of 10 animals and bars represent the SEM.

*P. berghei* ANKA is an established model of mouse cerebral malaria (CM) that reproduces reasonably well the pathological features of human CM. *P. berghei* infections are lethal in mice resulting in early death (by day 6–12) in susceptible B6 strains due to CM or, in late death (between days 14 and 21) in resistant strains due to severe anemia [34], [35]. B6 and B6.Tyr^−/−^ mice were infected with *P. berghei* ANKA by injection of 10^6^ infected red blood cells, i.p. All mice, both B6 and B6.Tyr^−/−^, died before day 10 and developed signs of CM (Figure 2). No differences in survival were observed between the groups. In addition, B6 and B6.Tyr^−/−^ mice showed no significant differences in parasitemia, hemoglobin levels, body temperature or cytokine levels (Figure S2) during the course of the infection. Thus, it appears that the ability to produce melanin is not protective against severe CM.

### Melanization does not protect against chronic malaria infection

**Figure 3:**
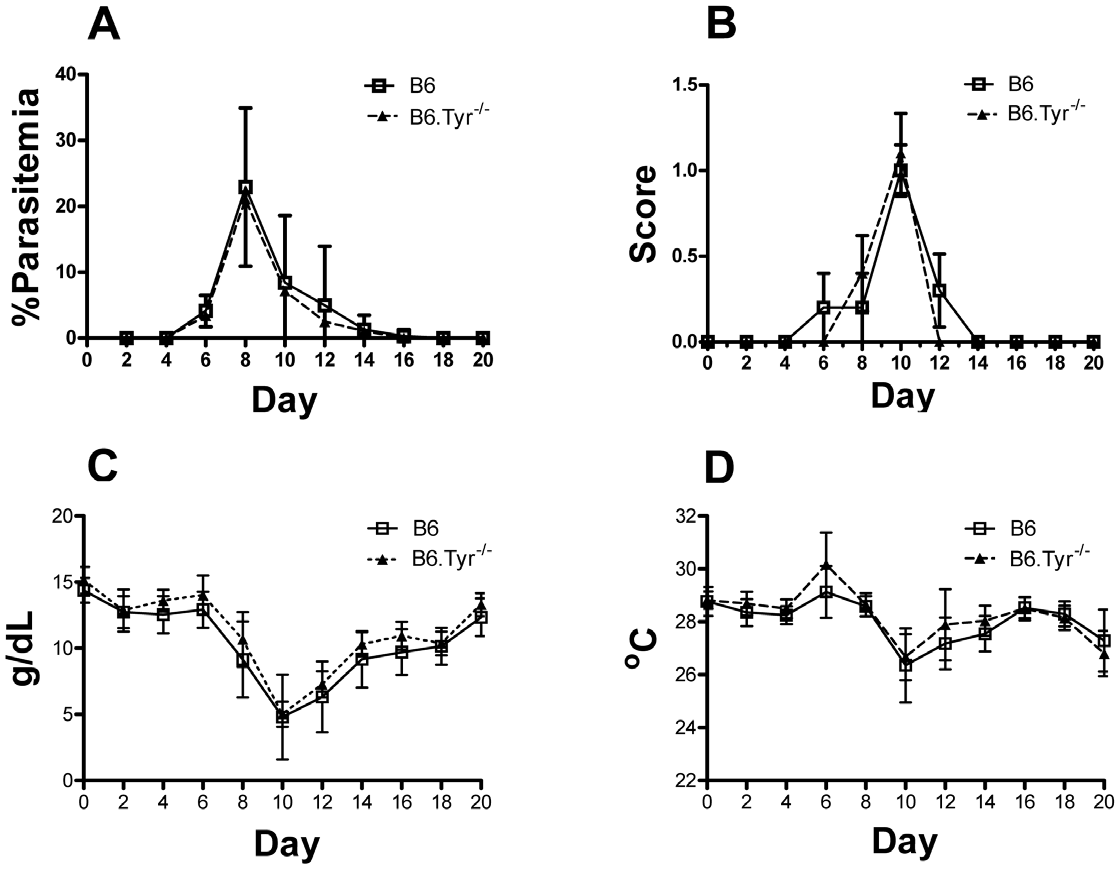
Effect of melanization on chronic malaria infection. (**A**) Parasitemias, determined by GIEMSA stained smears of tail blood, are given for B6.*Tyr−/−* and B6 *P. chabaudi* AS infected mice over time. (**B**) Clinical scores determined by the modified SNAP scoring system, are given. (**C**) Hemoglobin concentrations of B6.*Tyr−/−* and B6 infected mice are given. (**D**) Body temperature determined at the tail skin for both B6.*Tyr−/−* and B6 infected mice over time are given. Data showed represents the mean of 10 animals and bars represent the SEM.

To determine the effect of melanin deficiency on the course of a chronic malarial infection we infected B6 and B6.Tyr^−/−^ mice with *P. chabaudi* AS by injection of mice with 10^6^ infected red blood cells. *P. chabaudi* AS causes a biphasic infection characterized initially by acute parasitemia that peaks around day 7, followed by chronic low-level infection. The immune response during the first phase is controlled both by an innate system response as well as a Th1 response [36], [37] while the control and clearance of the chronic blood infection during the second phase requires both TH1 and TH2 CD4^+^ cells and B cells [36], [38]. We followed the *P. chabaudi* infected mice for 21 days and observed no difference between B6 and B6.Tyr^−/−^ mice in all parameters analyzed including survival, parasitemia, clinical score, hemoglobin levels, body temperature and leukocyte, neutrophil and lymphocyte numbers. (Figure 3, Figure S3). Thus, we concluded that the ability to produce melanin did not affect the course of chronic malaria infections.

### Melanin treatment decreases parasitemia but does not protect against cerebral malaria

**Figure 4:**
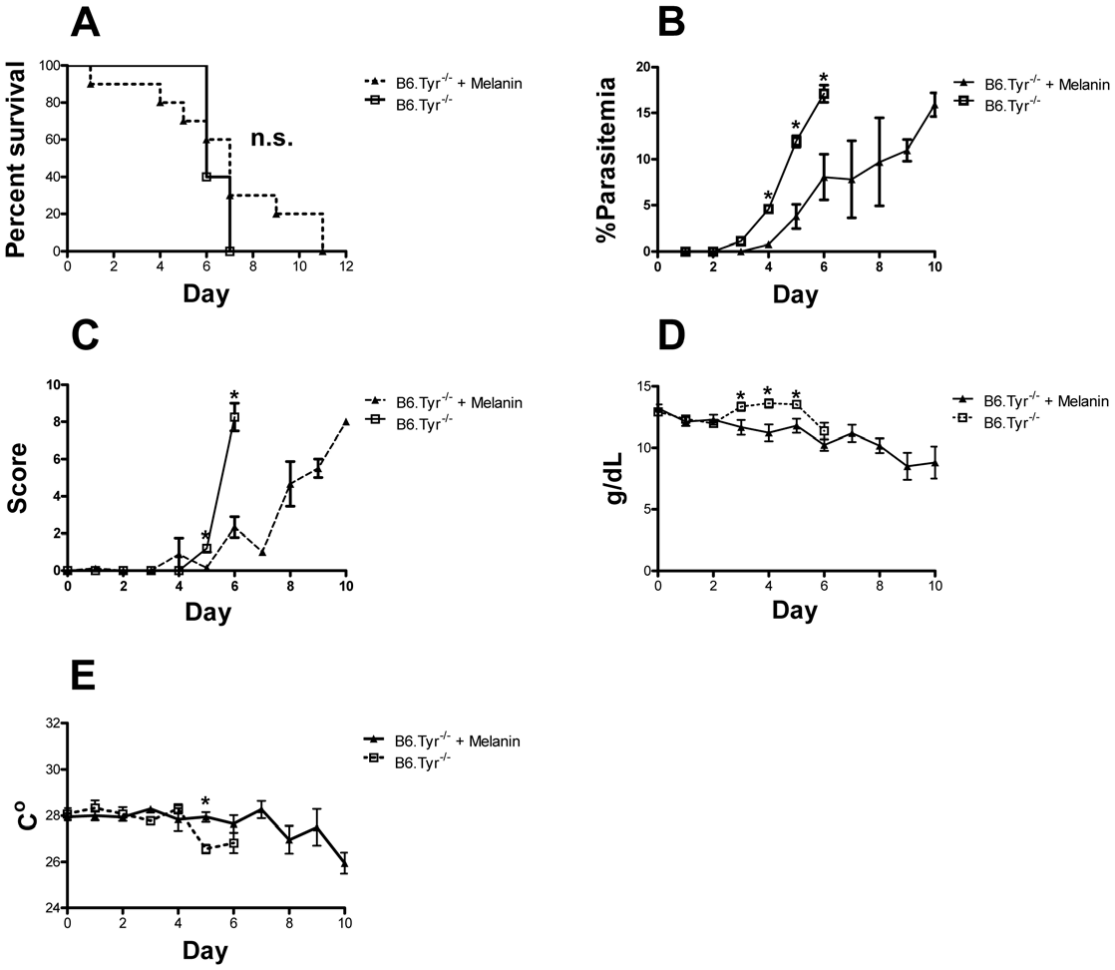
Effect of melanin treatment on cerebral malaria. (**A**) The percent of melanin treated and untreated B6.Tyr−/− *P. berghei* ANKA infected mice that survived over time is given in Kaplan-Meier curves. Death was defined as a parasitemia ≥60%, moribundity or death (P = 0.33). (**B**) Parasitemias, determined by GIEMSA stained smears of tail blood, are given for B6.*Tyr−/−* treated and untreated infected mice over time. (**C**) Clinical scores determined by the modified SNAP scoring system are given. (**D**) Hemoglobin concentrations of B6. *Tyr−/−* treated and untreated infected mice are given. (**E**) Body temperature determined at the tail skin for B6.*Tyr−/−* treated and untreated infected mice over time are given. Data showed represents the mean of 10 animals and bars represent the SEM.

Previous studies have shown that melanin in high doses can affect the secretion of multiple cytokines, including TNF-α, IL-1β, IL-6 and GM-CSF [39]. To investigate the effects of high doses of melanin on the course of malaria CM infections, we treated *P. berghei ANKA* infected B6.Tyr^−/−^ mice with daily doses of melanin (25 mg/kg) in saline i.p. or with saline alone. B6.Tyr^−/−^ mice receiving melanin showed a delayed increased in parasitemia (P<0.05) and better clinical scores but did not better survive the infection as compared to controls B6.Tyr^−/−^ mice receiving saline (P = 0.58) (Figure 4). On day five post-infection, melanin treated mice had higher platelet counts than controls (P<0.001) (Figure S4), and the number of platelets correlated positively with survival (Spearman Rho = 0.64, p = 0.002). On the other hand, melanin treatment caused animals to have a steeper decrease in hemoglobin levels, especially from day 3 to day 5 post-infection (P<0.05). The fact that melanin treated mice did not survive the infection any better than control B6 mice suggests that, albeit somewhat beneficial, melanin treatment is not sufficient to change the course of the disease in mice (Figure 4).

### Vitamin D does not protect against cerebral malaria

**Figure 5:**
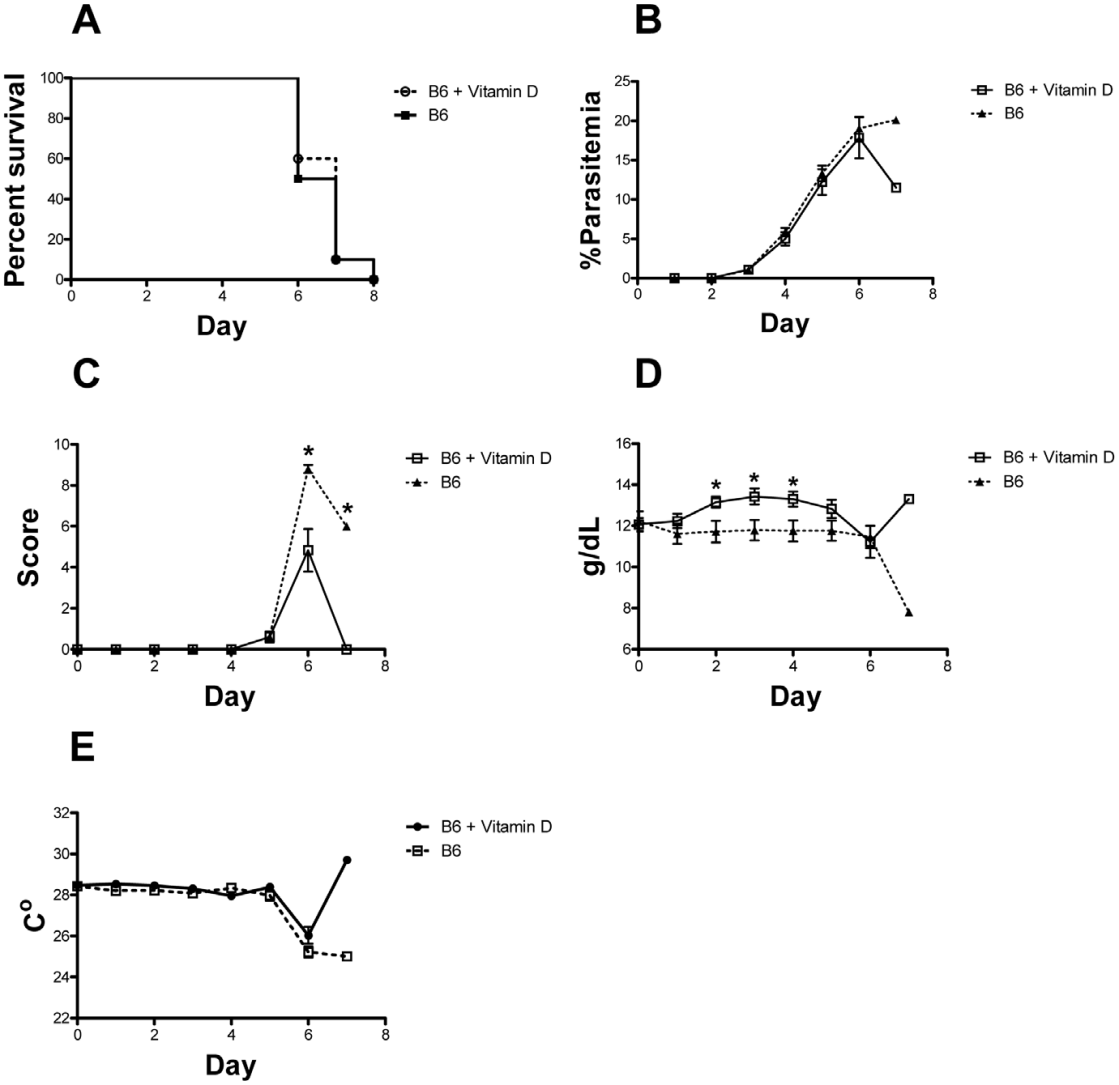
Effect of vitamin D3 treatment on cerebral malaria. (**A**) The percent of treated and control *P. berghei* ANKA infected B6 mice that survived over time is given in Kaplan-Meier curves. Death was defined as a parasitemia ≥60%, moribundity or death (P = 0.76). (**B**) Parasitemias, determined by GIEMSA stained smears of tail blood, are given for treated and control B6 mice over time. (**C**) Clinical scores determined by the modified SNAP scoring system are given. (**D**) Hemoglobin concentrations determined on tail blood are given. (**E**) Body temperature determined at the tail skin for treated and control B6 mice over time are given. Data showed represents the mean of 10 animals and bars represent the SEM.

Because skin melanization is an important factor in cutaneous vitamin D biosynthesis, we tested if vitamin D deficiency influenced the pathogenesis of cerebral malaria. We also supplemented the diets of mice with vitamin D injections i.p. every other day (0.5 µg/kg), starting 3 days before infection with *P. berghei* ANKA. We did not observe any difference in survival between treated and untreated mice with all mice dying by day 8 (Figure 5A). Also, we did not observe any differences in parasitemia or body temperature during the course of infection suggesting that vitamin D, at the levels used in this study, does not influence the immune response to malaria (Figure 5B and 5E). Animals receiving vitamin D injections had better clinical scores at days 6 and 7 post infection and had higher hemoglobin levels than controls but this effect was apparently not enough to reduce mortality (P = 0.76).

**Figure 6:**
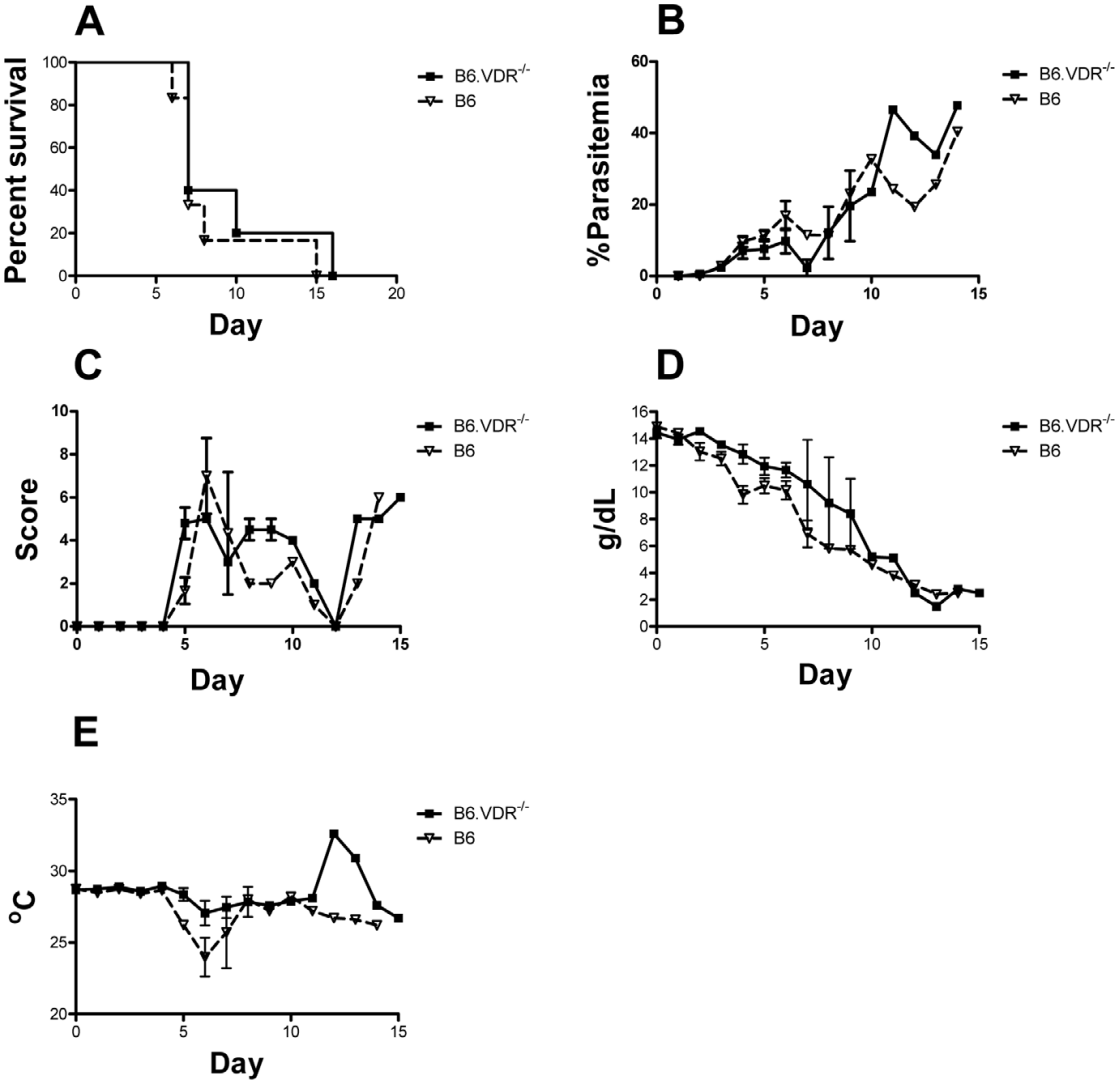
Effect of VDR knockout on cerebral malaria. (**A**) The percent of *B6* and B6.VDR−/− *P. berghei* ANKA infected mice that survived over time is given in Kaplan-Meier curves. Death was defined as a parasitemia ≥60%, moribundity or death (P = 0.38). (**B**) Parasitemias, determined by GIEMSA stained smears of tail blood, are given for B6.*VDR−/−* and B6 infected mice over time. (**C**) Clinical scores determined by the modified SNAP scoring system are given. (**D**) Hemoglobin concentrations of B6.*VDR−/−* and B6 infected mice are given. (**E**) Body temperature determined at the tail skin for both B6.*VDR−/−* and B6 infected mice over time are given. Data showed represents the mean of 10 animals and bars represent the SEM.

We also followed the course of *P. berghei* ANKA infections in wild type B6 mice and in mice deficient in the vitamin D receptor (VDR) and B6.VDR^−/−^ mice. Because B6.VDR^−/−^ knockout mice require a special diet to prevent severe osteoporosis and death, we kept wild type B6 mice on the same diet. We observed no difference in the course of disease in B6 and B6.VDR^−/−^ mice (Figure 6). Most B6 and B6.VDR^−/−^ mice died within 9–10 days of infection and showed similar parasitemias, clinical scores, hemoglobin levels and body temperatures.

## Discussion

Our results provide evidence that melanization does not improve survival in severe malaria. Melanization did not improve survival against parasites that cause either CM or severe anemia and does not affect the course of chronic infections. Our data also suggests that neither vitamin D treatment nor vitamin D receptor deficiency affect the progression of cerebral malaria caused by *P. berghei* ANKA infections. Therefore the data suggest that melanin, neither directly nor through its indirect effect on vitamin D synthesis, improves survival in severe malaria in mice. High doses of vitamin D have been reported to decreased parasite growth and survival *in vitro* [40] but we did not observe similar effects when treating animals with lower doses. This is in agreement with previous studies in humans showing a lack of association between polymorphisms on the VDR gene and malaria infection [19]. Nevertheless, because we only tested the effects of one dose of vitamin D3 (and not of other forms) one cannot exclude completely an effect of vitamin D on malaria.

We also tested the effect of high doses of melanin on the progression of CM. Although the dose used was previously shown to reduce LPS induced TNF-α production [39] and we showed that high doses of melanin delayed increases in parasitemia and improved clinical scores, melanin treatment was not sufficient to increase survival in CM. The effect in parasitemia and clinical scores can be potentially explained by direct anti-parasite effect of melanin (as observed in mosquitoes) or by its free radical scavenging properties. CM is a complex disease caused in part by excessive free radicals generated as a response to free iron and hemoglobin [41], [42], [43] and, under certain conditions, free radical scavengers can block some of the deleterious effects of malaria [44], [45]. Interestingly, melanin treatment prevented the thrombocytopenia that was observed in B6 mice by day 5 post-infection. Changes in platelet counts are also common in human malaria infection, and the extent of reduction is a predictor of clinical outcome and severity of malarial infection in children [46]. Platelets are involved in the pathogenesis of cerebral malaria as they encourage the sequestration of infected red blood cells in the cerebral vasculature and in the innate response to the parasite [47], [48] and, in agreement with the literature, our data showed a positive correlation between platelet count and survival. Last, we have shown that melanin treatment does not affect survival in *P. berghei* ANKA infections, suggesting again that melanin does not affect the progression of murine CM. Taken together, our data suggests that melanization was not selected by malaria. In spite of that, one cannot exclude completely the role of melanin on the response to malaria, for the mouse and human immune system and skin are considerably different. For instance, human melanocytes are mainly found at the basal layer of the epidermis while mouse melanocytes reside in the hair follicle and the dermis [49], [50], pelage density is much higher in rodents than humans, and the stratum corneum of rodents is generally more permeable and fragile [51]. Furthermore mice are nocturnal animals with very small vitamin D needs, suggesting that melanin must play a different role in vitamin D metabolism in mice. Humans, on the other hand, are considered diurnal animals that synthesize vitamin D in the skin on exposure to UV light [52].

## Methods

### Ethics statement

All experiments were approved by the National Institute of Allergy and Infectious Diseases Animal Care and Use Committee.

### Animals

Male 8–10 week old C57BL/6, B6.VDR[KO] (strain B6.129S4-Vdr^tm1Mbd^/J, stock number 006133) and B6.TYR[KO] (strain B6(Cg)-Tyr^c-2J^/J, stock number 000058) mice were obtained from The Jackson Laboratories and allowed to acclimate in the animal facility until the experiment was performed. B6.VDR[KO] mice were maintained constantly on a 20% lactose diet (TD 96348, Harlan) to prevent the development of osteoporosis.

### Malaria infections and treatments

Mice were infected with 1×10^6^ *P. berghei* ANKA, 1×10^6^ *P. chabaudi* AS or 1×10^4^ *P. yoelii* 17XL infected red blood cells (iRBCs) i.p. Parasites were obtained from donor mice that were infected with thawed parasite stocks. Synthetic melanin was obtained from Sigma (M0418) and it was initially dissolved in 0.1 NaOH to solubilize it. The solution pH was adjusted to 7.0, it was centrifuged and then filtered through a 0.45 µM filter before injection. Animals were injected i.p. daily with 25 mg/kg of melanin solution or saline. Vitamin D was obtained from Sigma (Supelco Cholecalciferol (D3) Cat#47763) and it was first dissolved in 10 mL of 100% ethanol to make a stock solution (10 mg/mL). The stock was further diluted in 0.02% Tween 80 to make a working solution (0.1 µg/mL). Animals were injected with 0.5 µg/kg every other day, starting 3 days before infection. A similar dose of 1,25-dihydroxivitamin D_3_ was previously shown not to cause hypercalcemia but to induce splenocyte apoptosis and enhance sensitivity to other parasites [53]. For the experiment investigating the role of the vitamin D receptor (VDR) on the response to cerebral malaria, VDR knockout mice were kept on a rescue diet rich in calcium, phosphorus and lactose (Harlan laboratories, stock #TD.96348) to avoid osteoporosis. 10 animals were used per group in all experiments. In all cases, parasitemia in infected mice was quantified by examining Giemsa stained thin blood smears. Hemoglobin concentration was measured daily with a HemoCue Hb 201+ using blood from the tail tip (<10 µL/day). Mice that reached 60% parasitemia or that become moribund were euthanized. All mice were evaluated daily for the presence of clinical signs of severe malaria using a simple scoring adapted from the SNAP scoring system [54]. Animals were scored by evaluating 5 categories: interactions/reflex, cage grasp, visual placing, gait/posture/appearance, and capacity to hold their body weight on a baton. Each category was divided in 3 levels, varying from 0 to 2, where 0 represented normal individuals and 2 the worst case for that particular parameter. Hematology parameters were determined on day 0 and 5 using a Hemavet 950 FS system (Drew Scientific).

### Statistics

When applicable, statistical analysis of survival was by Log-rank (Mantel-Cox) test. The survival curves were calculated using the Kaplan-Meier estimates and the survival curves were compared by the log-rank test. Continuous variables were analyzed with the two-sample t-test, where the Bartlett's test for equal variances was used to determine whether the use of a pooled variance or unequal variances was appropriate. Two-sided *P* values were reported in the text. *P* values were considered significant if *P*<0.05.

### Cytokine Quantification

The levels of cytokines in serum samples were quantified using the Q-Plex Mouse Cytokine Kit (Quansys Biosciences) according to manufacturer's instructions.

## Supporting Information

Figure S1 **Melanization does not change blood composition in response to severe malaria anemia.** Animals were infected with 1×10^4^ *P. yoelii 17XL* iRBCs on day 0. Hematological parameters were determined in whole blood. White blood cells (WBC), Red Blood Cells (RBC), Hemoglobin (Hb), Platelets, Neutrophils, Lymphocytes and Hematocrit are shown. Data showed represents the mean of 10 animals and bars represent the SD of the mean.
(TIF)

Figure S2 **Melanin does not alter the cytokine profile in response to cerebral malaria.** Cytokine levels before (day 0) and 5 days after infection using 1×10^6^ *P. berghei* ANKA iRBCs are shown. Data showed represents the mean of 10 animals and bars represent the SD of the mean.
(TIF)

Figure S3 **Melanization does not alter blood composition in response to chronic malaria.** Animals were infected with 1×10^6^ *P. chabaudi* iRBCs on day 0. White blood cells (WBC), Red Blood Cells (RBC), Hemoglobin (Hb), Platelets, Neutrophils, Lymphocytes and Hematocrit are shown. Data showed represents the mean of 10 animals and bars represent the SD of the mean.
(TIF)

Figure S4 **Effect of melanin injections on blood composition in B6.Tyr−/− animals.** Animals were infected with 1×10^6^ *P. berghei ANKA* iRBCs on day 0. White blood cells (WBC), Red Blood Cells (RBC), Hemoglobin (Hb), Platelets, Lymphocytes, Neutrophils, Hematocrit and the linear regression of survival on platelet counts are given. The dashed lines represent the 95% confidence interval about the regression line. Data showed represents the mean of 10 animals and bars represent the SD of the mean.
(TIF)
