# Supplementary figures and images for: Testing in Mice the Hypothesis That Melanin Is Protective in Malaria Infections
Source: PLoS One. 2012 Jan 5;7(1):e29493. doi: 10.1371/journal.pone.0029493 (PMC3252320; doi:10.1371/journal.pone.0029493)

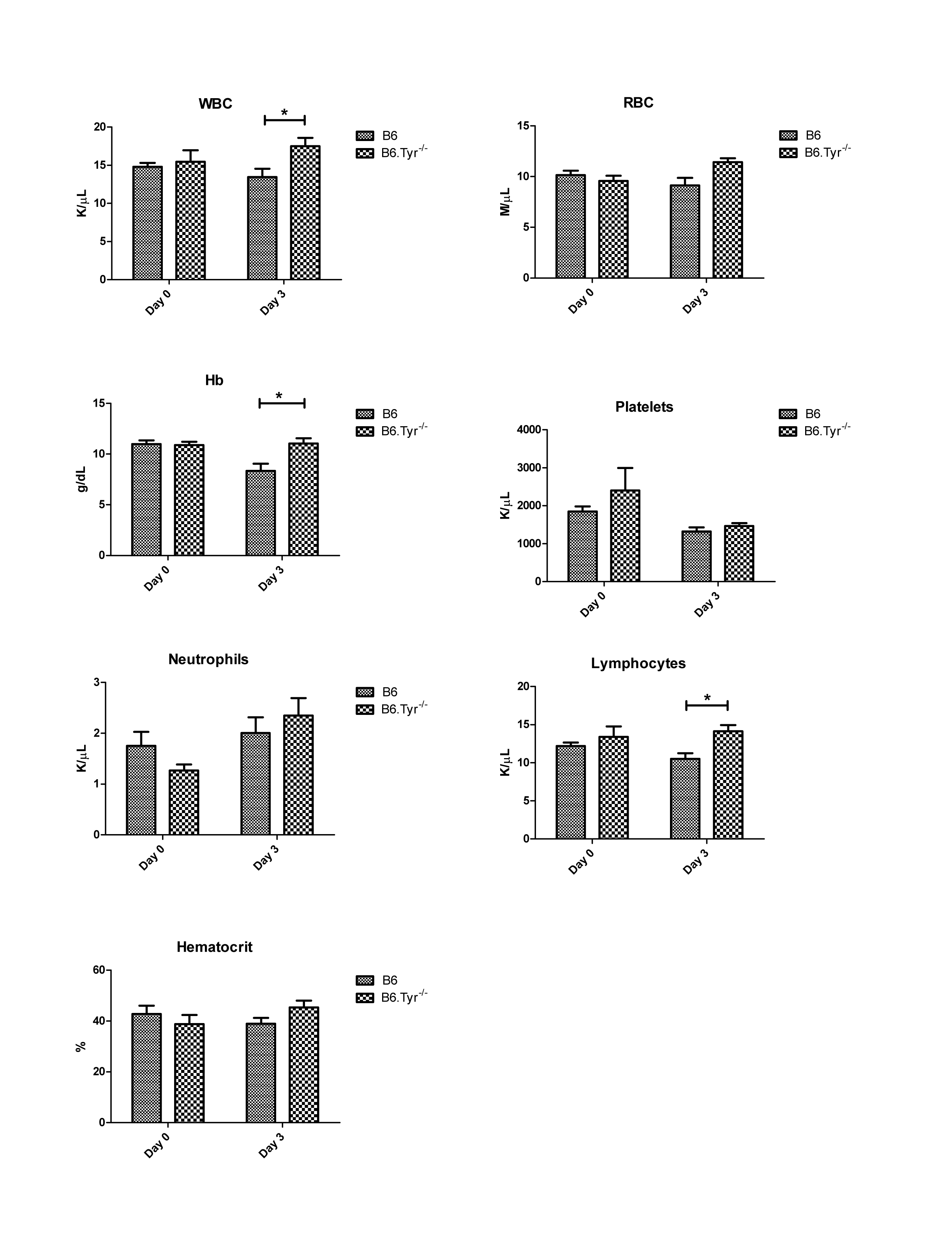

Supplement: Figure S1 — Melanization does not change blood composition in response to severe malaria anemia. Animals were infected with 1×10^4^ P. yoelii 17XL iRBCs on day 0. Hematological parameters were determined in whole blood. White blood cells (WBC), Red Blood Cells (RBC), Hemoglobin (Hb), Platelets, Neutrophils, Lymphocytes and Hematocrit are shown. Data showed represents the mean of 10 animals and bars represent the SD of the mean. (TIF) [file pone.0029493.s001.tif]

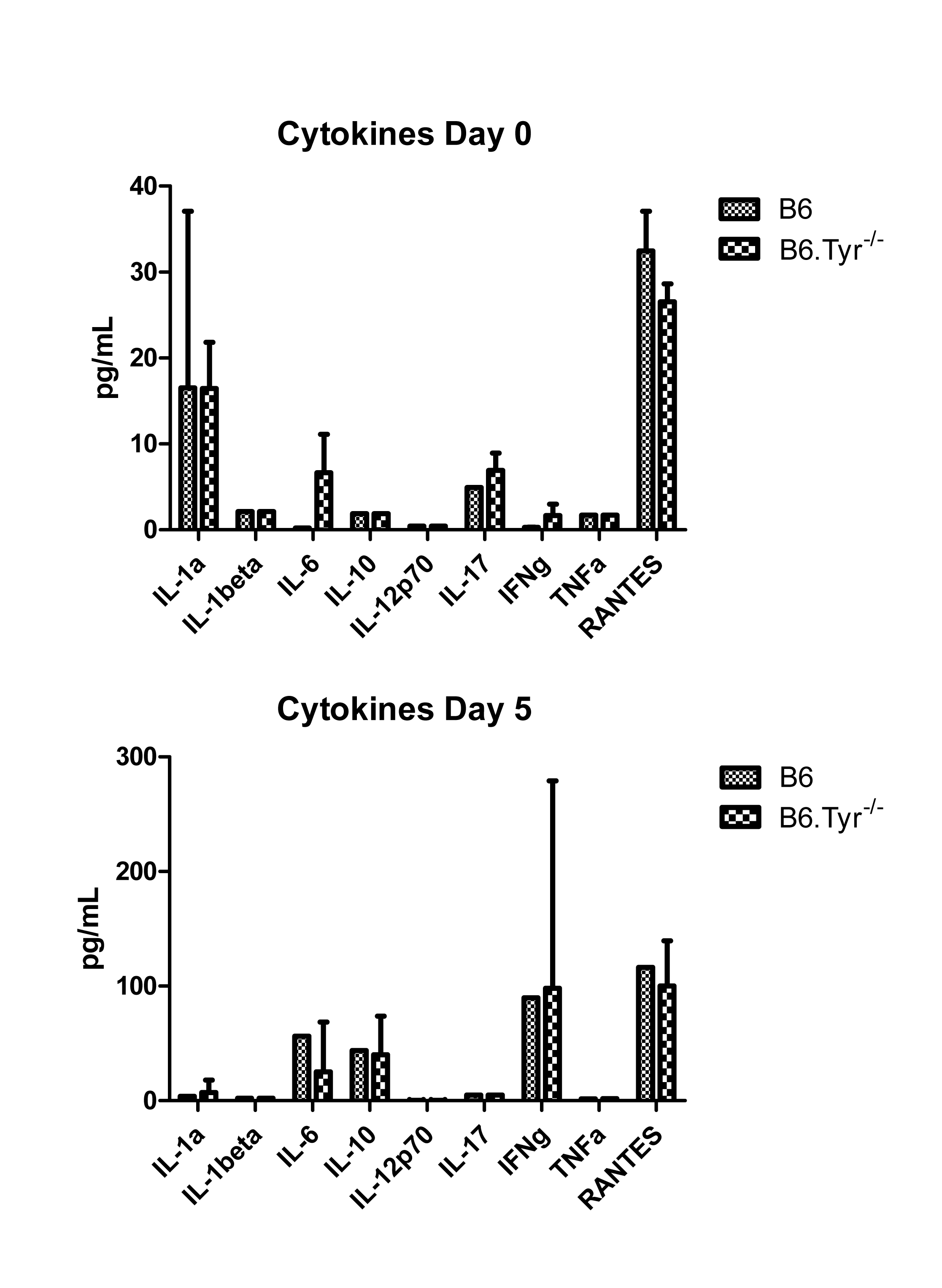

Supplement: Figure S2 — Melanin does not alter the cytokine profile in response to cerebral malaria. Cytokine levels before (day 0) and 5 days after infection using 1×10^6^ P. berghei ANKA iRBCs are shown. Data showed represents the mean of 10 animals and bars represent the SD of the mean. (TIF) [file pone.0029493.s002.tif]

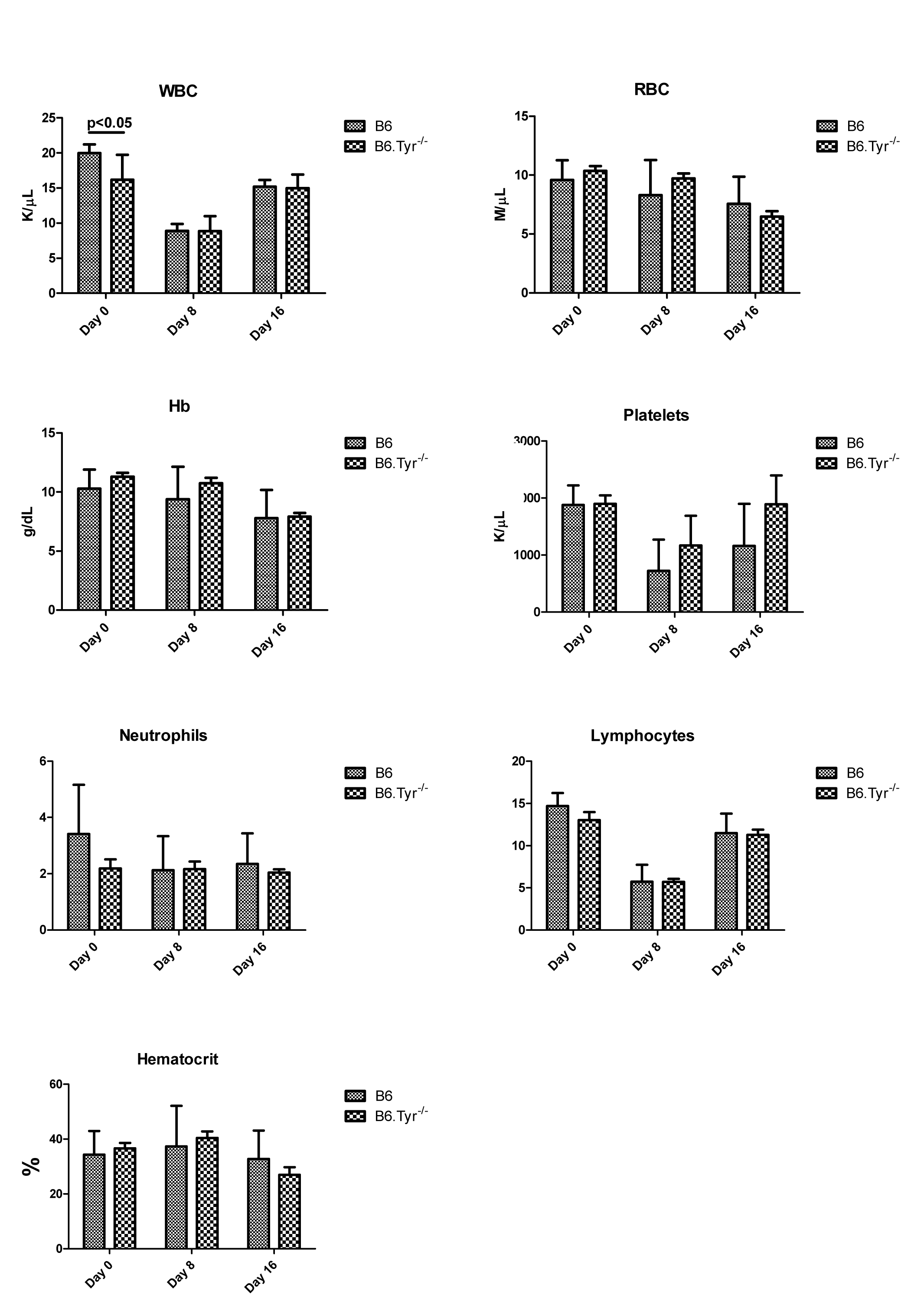

Supplement: Figure S3 — Melanization does not alter blood composition in response to chronic malaria. Animals were infected with 1×10^6^ P. chabaudi iRBCs on day 0. White blood cells (WBC), Red Blood Cells (RBC), Hemoglobin (Hb), Platelets, Neutrophils, Lymphocytes and Hematocrit are shown. Data showed represents the mean of 10 animals and bars represent the SD of the mean. (TIF) [file pone.0029493.s003.tif]

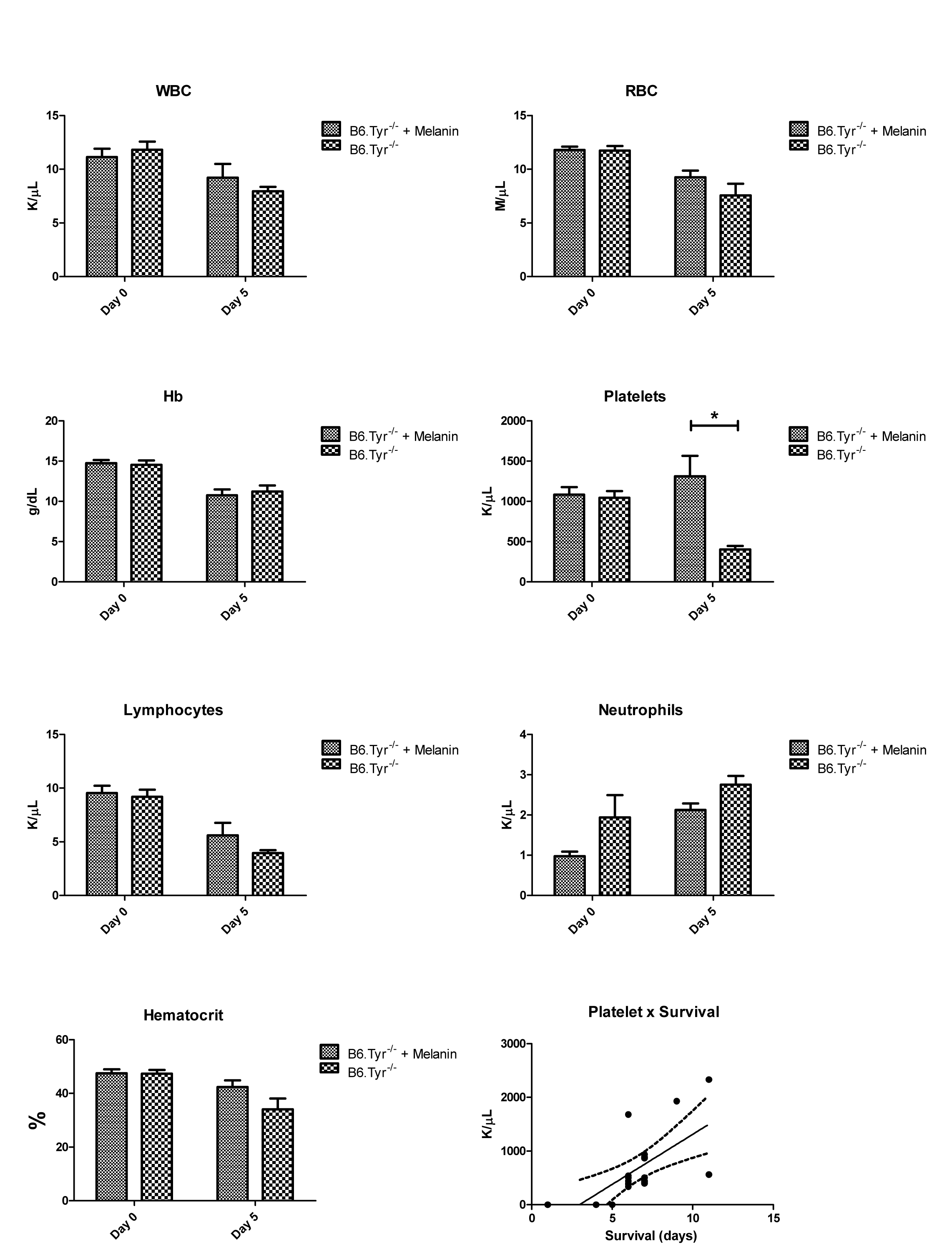

Supplement: Figure S4 — Effect of melanin injections on blood composition in B6.Tyr−/− animals. Animals were infected with 1×10^6^ P. berghei ANKA iRBCs on day 0. White blood cells (WBC), Red Blood Cells (RBC), Hemoglobin (Hb), Platelets, Lymphocytes, Neutrophils, Hematocrit and the linear regression of survival on platelet counts are given. The dashed lines represent the 95% confidence interval about the regression line. Data showed represents the mean of 10 animals and bars represent the SD of the mean. (TIF) [file pone.0029493.s004.tif]
